# Environmental impact assessment and monitoring of genetically modified trees

**DOI:** 10.1186/1753-6561-5-S7-O59

**Published:** 2011-09-13

**Authors:** Fernando Gallardo, Lucia Ionita, Marja Ruohonen-Lehto, Antoine Harfouche, Stefano Biricolti, Wout Boerjan, Boet Glandorf, Lise Jouanin, Matthias Fladung, Cristina Vettori

**Affiliations:** 1 Departamento de Biología Molecular y Bioquímica, University of Málaga, 29071 Málaga, Spain; 2 Department Genetics, Forest Research and Management Institute, 077190 Ilfov, Romania; 3 Finnish Environment Institute, 00251 Helsinki, Finland; 4 Department of Forest Environment and Resources, University of Tuscia, 01100 Viterbo, Italy; 5 Dipartimento di Ortoflorofrutticoltura, University of Florence, 50019 Sesto Fiorentino, Italy; 6 VIB Department of Plant Systems Biology and, University of Gent, 9052 Gent, Belgium; 7 National Institute for Public Health and the Environment, 3720BA Bilthoven, Netherlands; 8 UMR1318 INRA-AgroParisTech Versailles, Institute Jean-Pierre Bourgin, 78026 Versailles, France; 9 Institute of Forest Genetics, Johann Heinrich von Thünen Institute, D-22927 Grosshansdorf, Germany; 10 Consiglio Nazionale delle Ricerche, Istituto di Genetica Vegetale, 50019 Sesto, Fiorentino, Italy

## 

Transgenic biotechnology can assist forest tree improvement programs but it may also raise environmental safety concerns. The environmental effects of genetically modified transgenic trees (GMTs) have been studied in many countries during the last 15 years. Today there is an urgent need of putting together this scattered knowledge to build-up a European knowledge platform for addressing GMTs in plantations. The main aims of Working Group 2 (WG2) of the European Cooperation in Science and Technology (COST) Action FP0905 “Biosafety of transgenic forest trees” (http://www.cost-action-fp0905.eu/) are (1) to discuss, based on scientific facts, whether current containment strategies are appropriate or need to be improved for GMTs, (2) to define a common protocol to track the transgene from the laboratory to the final product, and (3) to assess the possible impacts of GMTs on the environment. The potential risks of GMTs, the fate of recombinant material and the potential relevance of recombinant genes on plantÂ´s omics are other main aspects to be considered and compared to similar processes with endogenous genes in conventional breeding. The group involves experts from public research, government and independent regulatory sectors across COST and non-COST member countries. The activities of this group have been organized into three Task Groups focusing on (1) risk assessment studies and guidance documents, (2) the monitoring of the transgenes and recombinant plant material, and (3) the impact of GMTs on exposed ecosystems. As a first step of WG2 activity, a database of guidance documents from national and transnational sources dealing with impacts and risk assessments of GMTs is being created to identify common and case-specific issues on biosafety. It is expected that the information gained will facilitate (1) a science-based understanding of the impacts of GMTs on the environment in comparison with that of traditionally tree breeding, and (2) future socio-economic and cost/benefits analyses of GMTs in plantations.

